# Revealing Genetic Dynamics: scRNA-seq Unravels Modifications in Human PDL Cells across In Vivo and In Vitro Environments

**DOI:** 10.3390/ijms25094731

**Published:** 2024-04-26

**Authors:** Ali T. Abdallah, Michael Peitz, Anna Konermann

**Affiliations:** 1 Cluster of Excellence Cellular Stress Responses in Aging-Associated Diseases (CECAD), 50931 Cologne, Germany; ali.abdallah@uni-koeln.de; 2 Institute of Medical Statistics and Computational Biology, Faculty of Medicine, University of Cologne, 50923 Cologne, Germany; 3 Interdisciplinary Center for Clinical Research, University Hospital RWTH, 52074 Aachen, Germany; 4 Institute of Reconstructive Neurobiology, Life and Brain Center, University Hospital Bonn, 53105 Bonn, Germany; 5 Department of Orthodontics, University Hospital Bonn, 53111 Bonn, Germany

**Keywords:** culture conditions, genetic markers, periodontal ligament cells, single-cell RNA-seq

## Abstract

The periodontal ligament (PDL) is a highly specialized fibrous tissue comprising heterogeneous cell populations of an intricate nature. These complexities, along with challenges due to cell culture, impede a comprehensive understanding of periodontal pathophysiology. This study aims to address this gap, employing single-cell RNA sequencing (scRNA-seq) technology to analyze the genetic intricacies of PDL both in vivo and in vitro. Primary human PDL samples (*n* = 7) were split for direct in vivo analysis and cell culture under serum-containing and serum-free conditions. Cell hashing and sorting, scRNA-seq library preparation using the 10x Genomics protocol, and Illumina sequencing were conducted. Primary analysis was performed using Cellranger, with downstream analysis via the R packages Seurat and SCORPIUS. Seven distinct PDL cell clusters were identified comprising different cellular subsets, each characterized by unique genetic profiles, with some showing donor-specific patterns in representation and distribution. Formation of these cellular clusters was influenced by culture conditions, particularly serum presence. Furthermore, certain cell populations were found to be inherent to the PDL tissue, while others exhibited variability across donors. This study elucidates specific genes and cell clusters within the PDL, revealing both inherent and context-driven subpopulations. The impact of culture conditions—notably the presence of serum—on cell cluster formation highlights the critical need for refining culture protocols, as comprehending these influences can drive the creation of superior culture systems vital for advancing research in PDL biology and regenerative therapies. These discoveries not only deepen our comprehension of PDL biology but also open avenues for future investigations into uncovering underlying mechanisms.

## 1. Introduction

Periodontal tissues are highly complex due to the multiplicity of interacting cells and extracellular matrices, the proximity of soft and mineralized tissues, and the constant challenges exerted by microbial and physical factors [1].

Among these tissues, the periodontal ligament (PDL) stands out as a specialized fibrous connective tissue located between the cementum and alveolar bone, consisting of a diverse cell population including fibroblasts, cementoblasts, osteoblasts, endothelial progenitor cells, macrophages, osteoclasts, and progenitor/stem cells [2]. The PDL not only supports teeth structurally but also plays vital roles in nutrition provision, tissue equilibrium, repair processes, and mechanical force sensing [3]. Although fibroblastic PDL cells are presumed to regulate PDL homeostasis and regeneration, the exact cellular subtypes responsible for these processes still remain to be elucidated [2,4].

PDL cells, with their spindle-shaped morphology, resemble gingival fibroblasts localized in close proximity, but exhibit distinct functional activities, performance in inflammatory settings, and regenerative patterns [5,6]. Differential gene expression likely underlies these functional distinctions, with studies showing significant genetic differences between PDL cells and gingival fibroblasts, but precise and in-depth data are not yet available [5].

Besides direct processing and analysis of explanted in vivo tissues, cell culture is the standard in vitro method for studying cellular behavior patterns, interactions, and signaling pathways. Although cultured cells are denoted as a homogeneous population when expanded under the same conditions as a kind of homogenizing agent and without further external influences, more recent approaches suggest heterogeneity evolving over time [7]. Furthermore, the type of cultivation has another potentially modulating influence on cells and their genetic profiles. Different cultivation techniques can influence cells and their genetic characteristics, with the widespread use of fetal bovine serum (FBS) in culture media serving to support cell attachment and growth, despite potential risks such as immune responses and alterations in cellular properties. Moreover, serum-based cultivation appears inadequate for clinical cell cultures and cell-based therapies [8]. Due to these long-standing practical, clinical, and ethical concerns about the use of serum, developing serum-free culture media formulations enabling both isolation and efficient cell expansion is crucial for maintaining cellular properties without these risks [9].

Nevertheless, various additional aspects certainly play a key role in the analysis of oral tissues as well, such as their heterogeneous nature and the different conditions to which the tissues are exposed to in vivo, both affecting their in vivo characteristics and impacting on their cultivation in vitro. Comprehending periodontal physiology and pathology is hindered by the intricate nature of these tissues coupled with challenges in in vitro culture, requiring a thorough examination of tissue composition, gene expression, and cellular changes between in vivo and in vitro environments to gain deeper insights into their molecular basis and regulatory mechanisms [10].

Next-generation sequencing (NGS) technology offers unparalleled efficiency, depth, and accuracy in transcriptome analysis, significantly advancing our comprehension of gene expression intricacies and regulatory networks within cells [11]. Initially, NGS was limited to mass sequencing of cell populations without enabling to account for cellular heterogeneity within populations, which was subsequently overcome by the development of single-cell RNA sequencing (scRNA-seq), allowing the dissection of genetic patterns at individual cell level, thus providing unprecedented insights into cellular diversity and complexity [12,13]. With the capability to study millions of cells simultaneously and decode their transcriptomes, scRNA-seq fundamentally changes the understanding of cellular biology by facilitating comprehensive molecular profiling and mathematical analysis of cellular expression states [14].

This study aims to utilize scRNA-seq to uncover the genetic pattern of PDL cells and investigate genetic changes induced by different culture conditions to provide insights into the cellular architecture and interactions within the PDL, addressing questions about cellular heterogeneity and genetic regulation in oral tissues.

Specifically, our objectives are:

1. To comprehensively characterize the genetic profile of PDL cells using scRNA-seq technology, providing a detailed insight of their complexity and individuality;
2. To compare genetic alterations in PDL cells cultured under serum-free and serum-containing conditions with those directly explanted from in vivo settings, aiming to determine whether culture procedures induce genetic changes and if serum supplementation has a distinct impact;
3. To identify and characterize distinct PDL cell types based on their highly expressed genetic markers, facilitating a deeper understanding of the cellular architecture and interactions within the PDL.

Through these objectives, we aim to advance our understanding of PDL biology and shed light on the impact of culture conditions on genetic expression patterns, ultimately contributing to broader insights into oral tissue regulation.

## 2. Results

### 2.1. Dimensionality Reduction and Clustering

**Figure 1:**
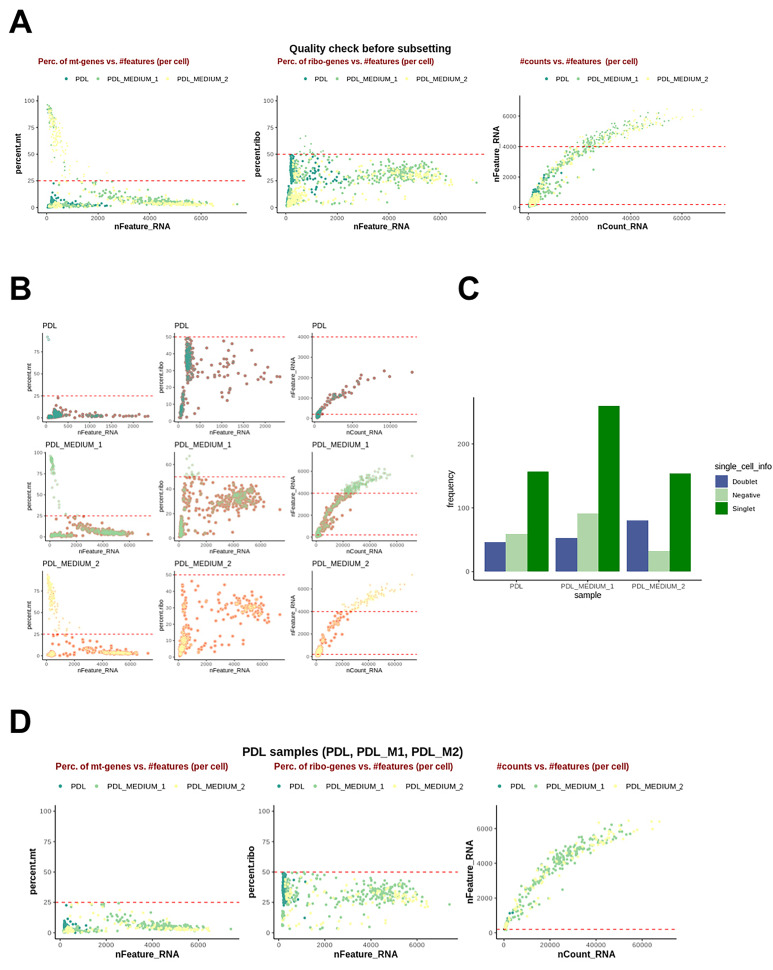
Quality control analysis for scRNA-seq data. (**A**) Combined overview of cells excluded and included for tissue samples of PDL, PDL cells cultured in serum-containing medium (PDL_M1), and PDL cells cultured in serum-free medium (PDL_M2). The left graph plots the percentage of mitochondrial genes in a cell (percent.mt), indicative of cell degradation and death, against the number of detected genes in this cell (nFeature_RNA). The center graph shows the percentage of ribosomal genes, related to cell cycle phases against the number of detected genes (nFeature_RNA). The right plots the number of detected genes (nFeature_RNA) against sequenced fragments (nCountRNA), highlighting potential doublets. (**B**) Sample quality before and after quality adjustment. Each dot represents a cell, with its position indicating the mitochondrial quality metrics. (**C**) Results of HTO demultiplexing, identifying demultiplexed singlet, doublets, and negative signal singlets. (**D**) Post-filtering status of PDL samples, showing the removal of doublets and other biased data to enhance the integrity of subsequent analyses.

**Figure 2:**
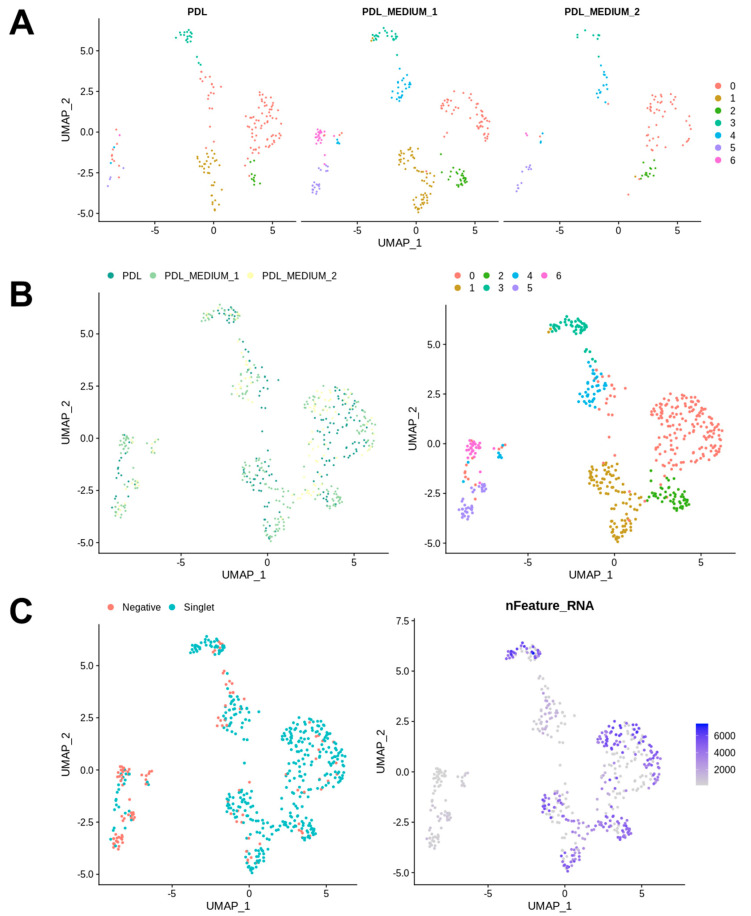
UMAP visualization for PDL cell analysis. This UMAP visualization presents the results of the clustering of PDL cells in vivo as baseline condition (PDL), PDL cells cultured in serum-containing medium (PDL_M1), and PDL cells cultured in serum-free medium (PDL_M2). (**A**) Clustering for each specimen into 7 color-coded clusters, showing the distinct cell populations within the PDL, PDL cells in serum-containing medium (PDL_M1), and PDL cells in serum-free medium (PDL_M2). The spatial arrangement of clusters within each graph signifies the segregation of distinct cell types, with cohesive cell populations consistently localized to specific regions across all three specimens. Each dot represents one cell. (**B**) Overview of clustering in 7 clusters, visually represented with distinct colors for each condition with regard to the sample types PDL, PDL_M1, and PDL_M2 on the left. The right-sided graph showcases the composition of clusters under different conditions altogether. The majority of cells were found in cluster 0, representing a fundamental cell state present in both in vivo PDL and cultured conditions. Cluster 1, with the second-highest number of cells, was mainly composed of PDL_M1 cells, suggesting serum-containing conditions support this cell state. Cluster 6 also exhibited notable presence of PDL_M1 cells, indicating serum influence. Clusters 4 and 5, induced by culture but not influenced by serum, had fewer cells and lower gene expression compared to clusters 0–3. (**C**) The left graph shows the 7 identified clusters based on the occurrence of demultiplexed cells (Singlet, turquoise) and unassigned cells (Negative, red), which could not be attributed to any donor. The right graph visualizes the clusters according to the number of genes expressed per cell, with color intensity indicating the level of gene expression per cell as depicted in the legend.

**Figure 3:**
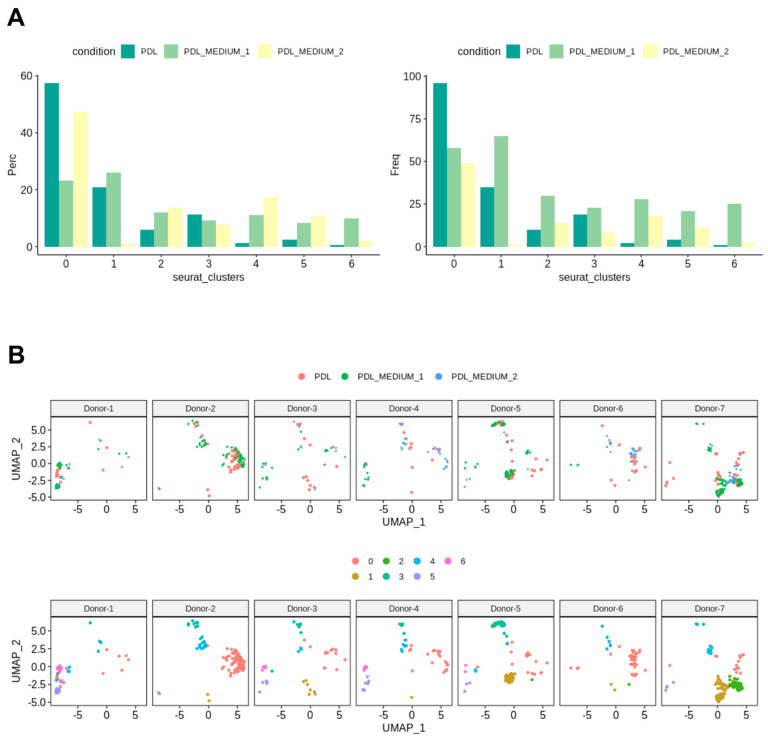
Analysis of cluster composition across conditions and donors. (**A**) Cell abundance analysis. Bar charts from the sample cell abundance analysis display the computed clusters by percentage (Perc) and absolute number of cells (Freq), representing the distribution of cells from different conditions (PDL, PDL_MEDIUM_1, and PDL_MEDIUM_2) across clusters. The left graph illustrates the percentage composition of cells (Perc) from each condition within each identified cluster. Each bar represents the proportion of cells from a specific condition, and the total percentage for each condition across all clusters adds up to 100%. The right graph shows the absolute number of cells (Freq) from each condition within the clusters, providing an absolute count that complements the percentage distribution. (**B**) Donor-specific cluster distribution: overview of individual donor samples and their individual distributions across samples (top row of UMAP plots) and clusters (bottom row of UMAP plots). Clusters 0, 3, and 4 were universally present, with variable intensities across donors. Clusters 1, 5, and 6 showed sporadic absence in some individuals. Cluster 2 was exclusive to donors 5, 6 and 7, notably dominant in donor 7. Each donor exhibited a distinct clustering pattern; for instance, emphasis on clusters 5 and 6 Donor 1, or remarkable prominence of cluster 1 in donor 7.

After quality control analysis for scRNA-seq data was conducted (Figure 1), a standard Seurat workflow was used to cluster cells after dimensionality reduction. Samples were integrated using Seurat’s standard integration workflow and analyzed independently. The analysis of the cell frequency in the calculated clusters was carried out and visualized in bar charts, as a low cell count in a cluster can influence the conclusions drawn and must be taken into account in the data analysis. The resulting UMAP grids and bar charts for PDL samples are illustrated in Figure 2 and Figure 3.

Figure 3A shows bar charts that depict the distribution of cells from the conditions PDL, PDL_MEDIUM_1, and PDL_MEDIUM_2 across all clusters. The left bar chart illustrates the percentage composition of cells from each condition within each identified cluster, with each bar representing the proportion of cells from one condition and the total percentage for each condition in all clusters sums to 100%. This visualization elucidates how each condition contributes to the total cell population within each cluster, with a high percentage in a particular cluster possibly indicating a strong association with that cluster’s cell subpopulation. The bar chart on the right displays the frequency of cells from each condition within each cluster, providing insights into the absolute size of each cluster in absolute terms and allowing for the comparison of the number of cells from each condition contributing to each cluster.

Our analysis identified seven distinct clusters. The majority of cells were generally found in cluster 0, which contains a significant proportion of cells from all conditions, namely PDL tissue (PDL), PDL cells in serum-containing medium (PDL_M1) and PDL cells in serum-free medium (PDL_M2), suggesting that it may represent a fundamental cell state or type present in both in vivo PDL and cultured conditions. Cluster 1 had the second-highest number of cells, but was predominantly composed of cells from PDL_M1, with PDL_M2 being almost absent and PDL tissue was only slightly represented. This might indicate that serum-containing conditions might support the cell state or type represented by this cluster. Remarkably similar, cluster 6 shows a notable presence of cells from PDL_M1 and was also almost solely generated due to cell culture in serum-containing medium. Clusters 4 and 5 were also exclusively induced by cell culture, but independent of serum influence, and exhibited low cell abundances. These clusters 4 to 6 also contained the most negative cells and those with the lowest number of expressed genes, whereas in contrast, the highest gene expression numbers were found in clusters 0–3 with only very few negative cells.

Figure 3B provides an overview of the donors, presenting for each specimen the individual cluster localization of their PDL tissue (PDL) and cultured PDL cells in serum-containing (PDL_Medium_1) and serum-free (PDL_Medium_2) medium, along with the presence of the seven identified clusters in their characteristic genetic profiles. It can be seen that for each donor the representation of cells from PDL tissue and from cell cultures is weighted differently and distributed within the clusters, thus reflecting the natural diversity among donors. However, the different representation and distribution pattern of the seven identified clusters among donors is notable. Cluster 0 was consistently represented in all donors, with donor 2 being the most pronounced, followed by donor 6. Clusters 3 and 4 were also found in all specimens, again with individual intensity depending on the donor. The other clusters were not found in all individuals, with clusters 1 and 5 not detectable in one donor each and cluster 6 not present in three donors. The pattern of cluster 2 was noteworthy, as it was exclusively present in three donors, namely donors 5, 6, and 7, and in the first two only represented by one cell, but contrarily constituted the second strongest cluster in donor 7. In addition, each donor showed an individual clustering pattern, with some observations being particularly noteworthy. Donor 1 had a strong portfolio with regard to clusters 5 and 6, which were rather underrepresented in the other donors, and exhibited hardly any cells in the other clusters, not even in the predominant cluster 0. Donor 2, on the other hand, was the strongest representative of cluster 0, but showed no cells at all for clusters 5 and 6, and thus exhibited exactly the opposite cellular focus compared to donor 1. Donors 3 and 4 presented a very analogous pattern with generally moderate but consistent cell presence in all clusters except cluster 2. Donor 5 was particularly conspicuous due to its strong representation of clusters 1 and 3, the latter being the most pronounced compared to all other individuals. Donor 6 showed a similar picture to donor 2 with a focus on cluster 0 and the absence of clusters 5 and 6, but with massively fewer cells overall. Donor 7 exhibited a particularly interesting and conspicuous pattern due to the already mentioned massive occurrence of cluster 2, and furthermore the strongest occurrence of cluster 1 in comparison to all other donors.

### 2.2. Marker Identification across Clusters

**Figure 4:**
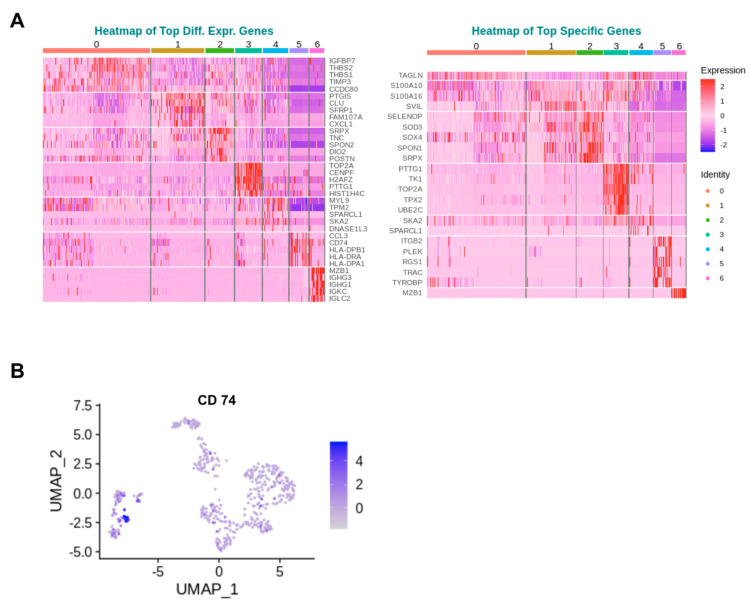
Genetic profiling and expression intensity in clustered cells. (**A**) Heat maps of the top differentially expressed genes (DEGs) and of the top-specific genes for each cluster within the PDL batch. DEG analysis identifies genes with notable expression differences between a specific cluster and others, highlighting potential functional markers across clusters. Specificity ranking pinpoints markers unique to each cluster, potentially providing clearer signatures of cellular identity through their distinct expression patterns. The heat maps are color-coded to reflect gene expression levels, with the scale bar indicating the range of expression. The cluster numbers are labeled at the top of the heat maps. (**B**) An illustrative UMAP plot showing the expression of *CD74* across clusters. The color scale on the right of the image indicates the respective expression intensities.

Cluster-specific markers were identified utilizing a two-pronged approach: differential expression analysis and specificity ranking. Differential expression analysis elucidates genes with notable expression differences between a specific cluster and the rest, shedding light on functional markers that might play significant roles across various clusters. Conversely, specificity ranking zeroes in on markers unique to each cluster, identified by their lower or non-existent expression in other clusters, thus possibly pinpointing more definitive signatures of cellular identity. This integrated methodology affords a nuanced understanding of cluster specificity, merging insights into both relative expression levels and distinct expression patterns of genes within clusters. Figure 4B exemplifies this by showcasing a Uniform Manifold Approximation and Projection (UMAP) plot for *CD74* expression, one of the top differentially expressed genes (DEGs) in cluster 5, demonstrating its expression profile across clusters 0–6. Here, the most highly expressed genes are considered per cluster, irrespective of whether they originate from PDL tissue or cultured PDL cells, with the goal of uncovering the identity of each cluster. The corresponding heatmaps are shown in Figure 4A.

The analysis revealed that the top three DEGs for cluster 0 were *IGFBP7*, which encodes insulin-like growth factor (IGF) binding protein 7, regulating the availability of insulin-like growth factors in tissues, stimulating cell adhesion and modulating IGF binding to its receptors; followed by *THBS2* and *THBS1*, whose proteins both mediate cell–cell and cell–matrix interactions. Regarding the most important specific genes, one gene was exclusively included in cluster 0, namely *TAGLN*. *TAGLN*, encoding transgelin, is a transformation- and shape-change-sensitive actin cross-linking/gelling protein found in fibroblasts and smooth muscle, and its downregulation may be an early and sensitive marker for onset of transformation.

In cluster 1, the three most highly expressed genes were *PTGIS*, encoding prostacyclin synthase, an enzyme catalyzing rearrangement of prostaglandin H2; followed by *CLU*, an extracellular molecular chaperone binding misfolded proteins to neutralize their toxicity and mediate cellular uptake through receptor-mediated endocytosis; and third, *SFRP1*, which can downregulate the Wnt signaling pathway, a crucial player in embryonic development, cell differentiation, and proliferation. The corresponding three most important specific genes were *S100A10* and *S100A16*, both belonging to the S100 family of proteins, which regulate a number of cellular processes such as cell cycle progression, differentiation, exocytosis, and endocytosis, whereby S100A10 in particular is additionally linked with the transport of neurotransmitters. The third-highest expressed gene was *SVIL*, implementing a potential role as a high-affinity link between the actin cytoskeleton and the membrane by recruitment of actin and other cytoskeletal proteins into specialized structures at the plasma membrane and in the nuclei of growing cells.

For cluster 2, the three most significant DEGs were *SRPX*, predicted to be an extracellular matrix structural constituent involved in cell adhesion; *TNC*, whose protein tenascin C is expressed in the extracellular matrix of various tissues during development, disease or injury and is upregulated by inflammation or at sites exposed to specific biomechanical forces; and in third place *SPON2*, which inter alia is predicted to enable antigen binding activity and cell adhesion. The three top-specific genes in cluster 2 were *SELENOP*, whose protein acts as an antioxidant in the extracellular space; secondly, antioxidant enzyme *SOD3,* and thirdly, *SOX4* functioning in the apoptosis pathway and mediating downstream effects of parathyroid hormone (PTH) and PTH-related protein (PTHrP) in bone development.

The three priority DEGs in cluster 3 were *TOP2A*, whose nuclear enzyme controls and modifies the topologic states of DNA during transcription; *CENPF*, involved in chromosome segregation during cell division and in orientation of microtubules to form cellular cilia, and *H2AFZ*, encoding the thermosensory response mediator histone H2AZ. The top-specific genes were *PTTG1*, which is inter alia involved in stimulating expression of basic fibroblast growth factor, *TK1*, which codes for the cell cycle-regulated enzyme thymidine kinase 1 with importance for nucleotide metabolism and, analogous to the top DEGs, again *TOP2A*.

Looking at cluster 4, the top three DEGs represent *MYL9*, whose encoded protein is a myosin light chain regulating muscle contraction, the *TMP2*-related protein tropomyosin 2, which binds to actin filaments in muscle and non-muscle cells and plays a central role in calcium-dependent regulation of striated muscle contraction as well as *SPARCL1*, which interacts with the extracellular matrix in order to create intermediate states of cell adhesion as well as being associated with the repair of muscle damage. Similar to cluster 0, there were exclusively two top-specific genes in cluster 5, namely *SKA2*, which enables microtubule binding activity and, congruent with the DEGs of the cluster, again *SPARCL1*.

The first three DEGs in cluster 5 were inflammatory cytokine *CCL3* as well as *CD74* and *HLA-DPB1*, the latter two being involved in antigen presentation via MHC II. The corresponding top-specific genes were *ITGB2*, whose coding surface protein integrin β-2 establishes contacts between cells as well as contacts to the tissue portion between cells, *PLEK*, which is involved in actin cytoskeleton organization among various other processes and *RGS1*, which regulates G protein-coupled receptor signaling cascades.

For cluster 6, the top three DEGs were *MZB1*, which is involved in positive regulation of cell population proliferation and is supposed to exert its effect via action of molecular chaperones, as well as, in second and third place, Immunoglobulin Heavy Constant Gamma 3 and 1 (*IGHG3,1*). These are thought to enable antigen binding activity and immunoglobulin receptor binding activity and to be involved in several processes including activation of the immune response, defense response to other organisms, and phagocytosis. The only top-specific gene present for cluster 6 was again *MZB1*.

### 2.3. Conservation Analysis across Donors

**Figure 5:**
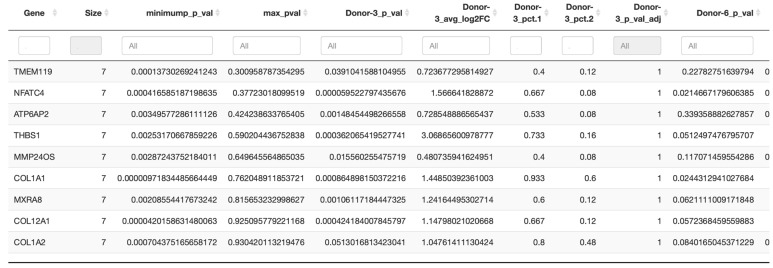
Conserved markers of cluster 0. Results of the conservation analysis across all 7 donors showing the top 10 conserved markers sorted by the most conservative significance assumption (max_pval) of cluster 0. Top 10 genes are listed in the left column.

In the next step, a conservation analysis was performed across all donors, pooling all samples together, in order to identify the top 10 conserved markers sorted by the most conservative significance assumption (max_pval). Since cluster 0 was expressed in all donors and also contained the absolute majority of PDL cells, it was used to identify the genes of this complex that are conserved across all donors and thus represent the characteristic of this cluster. As exhibited in Figure 5, the most significantly conserved marker of cluster 0 was *TMEM119*, whose encoded Transmembrane Protein 119 is involved in the positive regulation of bone mineralization as well as osteoblast differentiation and proliferation. The other genes of interest were primarily *COL1A1* and *COL1A2*, which encode type I alpha-1 collagen and type I alpha-2 collagen, with type I fibrillar collagen being the most abundant type of collagen in the body and *COL12A1*, whose protein is a type XII collagen and is responsible for cell adhesion, collagen degradation and collagen fibril organization, among other functions.

Finally, also of interest was *MXRA8*, whose coding transmembrane protein can modulate the activity of various signaling pathways, including inhibiting osteoclastogenesis downstream of TNFSF11/RANKL and CSF1, possibly attenuating signaling via integrin ITGB3 and MAP kinase p38.

### 2.4. Pseudotime Analysis

**Figure 6:**
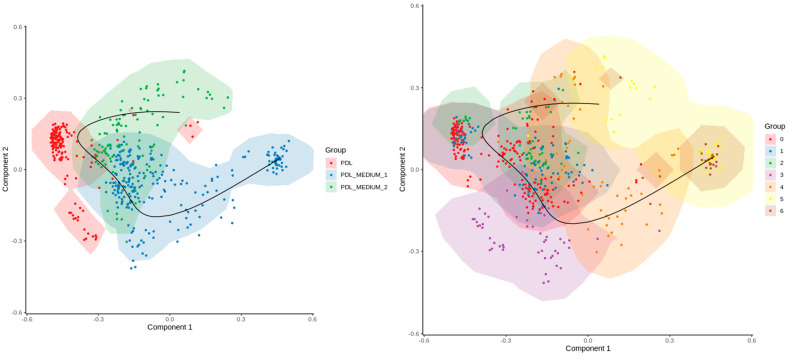
Pseudotime trajectory analysis. Results of the pseudotime analysis plotted in a two-dimensional space defined by component 1 and component 2 of SCORPIUS’s own dimensionality reduction algorithm. The left plot features a trajectory, delineated by a black line, which indicates a potential developmental path or differentiation process. It suggests that cells of conditions PDL_M1 and PDL_M2 originate from PDL and undergo certain changes due to the culture.

Finally, to elucidate the developmental progression of cells, we conducted a pseudotemporal trajectory inference using single-cell data. The results are plotted in a two-dimensional space defined by component 1 and component 2 (Figure 6). Using this technique in single-cell transcriptomics, the pattern of the dynamic process that cells undergo developmentally in temporal sequence was determined, subsequently arranging cells based on this progression. In the left plot, the trajectory presented as a black line indicates the cellular developmental path and reveals that the cells of conditions PDL_M1 and PDL_M2 originate from PDL and furthermore undergo certain changes due to the culture conditions. In the right plot, the trajectory contextualizes the cluster progression states in temporal sequence, indicating that the early formations are clusters 0–3, from which the clusters 4–6 are then differentiated.

## 3. Discussion

In this study, we used single-cell RNA sequencing to identify and characterize different fibroblast populations in the PDL. Additionally, we detected rare subsets of PDL cell populations emerging during cell culture and analyzed their distinct genetic profiles under serum-containing and serum-free conditions. Our findings indicate that some fibroblast populations are naturally situated within the PDL tissue, while others respond specifically to their environment. The finding that certain genes consistently show expression within a specific cluster across all donors suggests a pivotal role for these genes in maintaining the structure and function of the PDL. In-depth analysis of this cluster in subsequent studies holds the potential to uncover PDL cell-characteristic markers, thereby advancing our understanding of periodontal biology. These discoveries hold promise for developing novel diagnostic tools and therapeutic approaches for improving periodontal health and treatment outcomes.

Our study represents a significant advancement in the field, as it is the first to combine both in vivo and in vitro data of the PDL from the same specimens for scRNA-seq analysis, and moreover examine in vitro cells cultured under serum-free and serum-containing conditions. The novelty of this comprehensive approach, which has not been previously undertaken, provides a broad and unparalleled view of the genetic profiles and functional dynamics of PDL cells in different environments. Through this innovative methodology, this study elucidates the intricate interplay between cellular responses and environmental factors within the PDL microenvironment at the single-cell level, gaining deeper insights into genetic distinctions and functional diversity of PDL cell populations.

Furthermore, the inclusion of multiple donors enabled exploration of individual-specific variations in PDL composition, adding another layer of complexity to the findings of PDL dynamics.

Our analyses revealed that the transition from in vivo to in vitro alone resulted in the formation of two new cell clusters with very characteristic features. It was extremely striking that the predominant genes in cluster 4 as one of these two clusters were MYL9 and TMP2, two markers for the regulation of muscle contraction, and SPARCL1, a gene associated with the repair of muscle damage [15]. Thus, under culture conditions, a fibroblastic population appears to emerge that corresponds to a myofibroblastic cell type. These findings are supported by another recent study on dermal fibroblasts, postulating a lineage plasticity with distinct differentiation pathways from fibroblasts to a specialized contractile myofibroblasts, specifically attesting to a dynamic nature of fibroblast identities during wound healing [16]. Furthermore, studies revealed that PDL cells can transform into myofibroblasts during orthodontic tooth movement by activating the RhoA/ROCK pathway as a result of the occurring mechanical strains and express the myofibroblast-specific marker α-smooth muscle actin due to biomechanical signal transduction in a stimulus-induced manner [17,18]. In order to understand which environmental influences evoke a cell cluster with predominant muscle-associated marker expression in this study, further intrinsic mechanisms must be investigated as cells were not exposed to any mechanical stimuli.

The second new cluster 5, exclusively evolved by the transition from in vivo to in vitro alone, was characterized by its first three DEGs comprising inflammatory cytokine *CCL3* as well as the MHC II antigen presentation key molecules *CD74* and *HLA-DPB1*, suggesting an activated cellular cluster type involved in the immune response. Previous studies have already shown that PDL cells possess immunomodulatory properties and secrete proinflammatory cytokines such as the proinflammatory *CCL3* discovered in our work, suggesting the possibility of further differentiation into immunocompetent cells capable of antigen presentation via MHC II [19,20].

The large genetic changes exclusively caused by the in vitro culture compared to cells in situ uncovered here have been found for other cell types as well [21,22]. This nature of metabolic plasticity depending on the environment must be taken into account in future in vitro studies intended to reflect the in vivo situation and at best be eliminated by more precise knowledge of the underlying causes.

In addition to the genetic changes and resulting formations of new clusters due to the fact of cultivation, our data also revealed that two further clusters developed exclusively upon presence of serum and were undetectable in serum-free conditions. The genetic profile of cluster 1 was dominated by markers for cell differentiation, proliferation, and cell cycle progression, which confirms the well-known extraordinary growth-inducing composition of factors in serum, but, as the comparison with in vivo cells shows, does not correspond to reality and thus has implications for the future development of in vitro models [23]. Cluster 6, as a second cluster exclusively evolving due to presence of serum, was characterized as well by a marker involved in the positive regulation of cell population proliferation and furthermore by markers enabling antigen as well as immunoglobulin receptor binding activity, which are additionally involved in activation of the immune response, defense response to other organisms, and phagocytosis. The immunomodulatory functions of PDL cells have already been described, but based on the data presented here, it is unclear to what extent FBS plays a role in artificially inducing these properties, especially in light of the fact that studies have already demonstrated the alteration of the cellular transcriptional profile by FBS [9,24]. FBS is a variable and undefined component of the medium with a complex composition and may contain generally unpredictable factors that are not yet fully understood.

Pseudotime analyses underline these results that PDL cells undergo a developmental path in culture as well as sequential transcriptional changes due to culture conditions compared to the PDL they were originating from, which is also reflected by cluster progression from more undifferentiated to specialized cell states. Notably, clusters 4–6 are further away along the inferred developmental trajectory. As these clusters primarily consist of cells cultured under specific conditions, this might indicate that these conditions drive cells towards a distinct differentiation path or induce stress responses that are not present or less pronounced in the earlier clusters. A further study of these differences and the nature of these cells could reveal some implications related to functional capabilities and limitations of these cells on the one hand or optimization of culture conditions to either promote or inhibit the formation of these later-stage clusters, depending on the desired outcome for cell culture or tissue engineering applications.

Our analyses further showed that the cells from PDL tissue and from cell cultures are differently weighted and distributed within the clusters in each donor, and furthermore, that each donor had a different pattern of representation and distribution of the seven identified clusters. To fully understand the biological implications of these cellular distributions of in vitro and in vivo cells within clusters, a further study will need to deeply investigate the gene expression profiles and functional annotations associated with each cluster separately. The differences in cluster composition between conditions are indicative of how the in vitro culture conditions affect the state of the cells, which is important for interpreting the relevance of in vitro studies to in vivo biology. The variability in cluster distribution between conditions could influence the generalization of results beyond the study, as it suggests that different culture conditions may lead to different cellular compositions. Throughout the entire span of research on periodontal structures, fibroblasts have eluded precise classification due to the lack of specific markers and have escaped distinct specification of their heterogeneous subpopulations, which was further complicated by partial overlaps and strong similarities of the same cell types.

The findings of this study hold significant clinical relevance, particularly in understanding the pathogenesis of periodontitis, as the identification of cluster-specific markers sheds light on the intricate molecular mechanisms underlying periodontal tissue homeostasis and dysfunction. Deficiency or dysregulation of certain genes, as highlighted by the differential expression analysis and specificity ranking, may contribute to the development and progression of periodontitis. For instance, downregulation of *TAGLN*, a gene encoding transgelin involved in actin cytoskeleton organization, may indicate early pathological changes associated with cellular transformation and shape alterations as characteristic features of periodontal tissue remodeling in response to inflammation and mechanical stress [25,26]. Furthermore, alterations in the expression of *SELENOP*, *SOD3* and *SOX4* in cluster 2 highlight potential disruptions in antioxidant defense mechanisms and apoptosis regulation, being critical for periodontal tissue homeostasis [27,28,29]. Moreover, the upregulation of inflammatory cytokines such as *CCL3* and immune response-related genes such as *CD74* and *HLA-DPB1* in cluster 5 suggests an enhanced immune response within the periodontal microenvironment as a hallmark of periodontitis-associated inflammation and tissue destruction [30,31,32]. Overall, the elucidation of cluster-specific markers and conservation patterns provides valuable insights into the molecular mechanisms underlying periodontitis pathogenesis. Targeting these genes and pathways in case of dysregulation may offer novel therapeutic strategies for the prevention and management of periodontal diseases, ultimately improving clinical outcomes and oral health quality.

Our results reveal for the first time that there are specific genes and resulting cell clusters in PDL culture whose expression underlies contextual aspects, as well as subpopulations that are independent of such environmental factors. These aspects could be optimally decoded with the scRNA-seq method used in this study, whereby the 10x Genomics protocol applied here, in contrast to other commercially available scRNA-seq protocols, enables the sequencing of thousands of cells simultaneously. This is done with a much lower read depth per cell and without the use of fluorescent markers to determine cell identity, making the 10x Genomics platform as particularly suitable for the detailed characterization of heterogeneous tissues such as the ones investigated here [33].

The study presents valuable insights into gene expression patterns and cell clusters in PDL cultures, albeit with limitations to be considered when looking at the results. While the scRNA-seq method employed is a recognized standard, reliance on a single method can introduce inherent biases, necessitating cautious interpretation. The study’s conclusions should be contextualized within the chosen methodology’s parameters, recognizing that different sequencing methods may yield differing results. Furthermore, the generalizability of the results may be limited by the specific characteristics of the PDL cultures used in this study. Factors such as donor variability, culture conditions, and number of passages could in principle influence gene expression patterns and cell behavior, potentially leading to biased or incomplete conclusions when relying exclusively on these data, urging careful extrapolation to other settings. Although the study identifies relevant genes and cell groups, their functional significance remains to be fully understood, prompting further validation through diverse approaches.

The results of our study lay the foundation for further analysis of the lineage relationships of PDL cell subpopulations as well as characterization of the factors determining their plasticity and fate change, thus paving the way for new state of the art therapeutic approaches to tissue regeneration and healing in the periodontium. Our present study provides a basic prerequisite for such scientific progress in the field of periodontal research.

## 4. Material and Methods

The study was performed according to the ethical principles of the World Medical Association Declaration of Helsinki. Informed consent was obtained from all individual human donors of the experimental material included in the study. The study has been independently reviewed and received Institutional Review Board (IRB) approval by the Ethical Committee of the University of Bonn (reference number 029/08).

### 4.1. Isolation and Sample Preparation of Primary PDL

Primary human PDL was achieved from periodontally healthy adult male (*n* = 2) and female (*n* = 5) donors with a mean age of 26.3 ± 11.2 undergoing extraction of erupted wisdom teeth (*n* = 7). Patient inclusion criteria based on panoramic radiographs and clinical examination were as follows: no systemic problems or medication affecting oral health; no need for an osteotomy to minimize the risk of injury to the PDL; teeth with completely developed root apices to ensure that no pulp tissue can contaminate the tissue harvest; and securing direct sample processing after extraction. Specimens were kindly provided by the Private Practice Dr. Vollmar, Wissen, Germany; the Private Practice Dr. Dr. Appel, Sankt Augustin, Germany; and the MEDECO Clinic Bonn, Germany. Primary PDL was explanted from the middle third of the root surface of the wisdom teeth. Enzymatic dissociations of PDL (*n* = 7) to obtain single-cell suspensions was performed by washing extracted teeth with DMEM medium, scraping off the PDL cells from the root surface and mincing them before incubating the small tissue pieces with 0.5 mL enzyme mix (12.5 μL collagenase/dispase and 5 μL DNase I) at 37 °C for 30 min. Residual tissues were disaggregated by gentle flushing until single-cell suspensions were obtained. Each sample was filtered through a 70 μm filter attached on 50 mL tubes. Filters were washed with 10 mL of medium and cells were centrifuged at 300× *g* for 5 min before resuspending them in 0.2 mL of medium. Viability testing and cell count analysis was performed using trypan blue and a hemacytometer. Specimens were cryopreserved by adding 10% DMSO into cell suspensions, storing samples at −80 °C for one day and then transferring them to −150 °C.

### 4.2. Primary PDL Cell Isolation and Culture

From the isolated primary human PDL (*n* = 7), one half of the material was processed directly for further analysis as described above, and the other half was used to study the differentiation of PDL cells under two different media culture conditions. Cells were grown in cell culture flasks (T75, CELLSTAR^®^ Greiner BioOne, Kremsmünster, Austria), one half in N2B27-PDLsf medium [34], a serum-free medium formulation for successful PDL cell cultivation over several passages, and one half in serum containing Dulbecco’s Modified Eagle Medium (DMEM)-based medium (sDMEM) supplemented with 10% heat inactivated fetal calf serum (Invitrogen). Cells were grown at 37 °C in a humidified 5% CO_2_ atmosphere and passaged after reaching confluence. Medium was supplemented with 1% Penicillin-Streptomycin (Gibco, Carlsbad, CA, USA) and 1% Plasmocin prophylactic (Invivogen, Toulouse, France) until passage 2. From passage 3, both media were used in the absence of Penicillin-Streptomycin and Plasmocin prophylactic. Culture-expanded cells were utilized for analyses at passage 3–4. The passaging of cells was performed with StemPro Accutase (Gibco) for 5–10 min at 37 °C and the dissociation was stopped by diluting the enzyme with medium.

### 4.3. Cell Hashing and Sorting from Single-Cell Suspension

Single-cell aliquots were thawed and supplemented with 5 mL of medium before centrifuging at 500× *g* for 5 min. After discarding the supernatants, cells were resuspended in 100 μL cell staining buffer (Biolegend, San Diego, CA, USA; cat # 420201). 5 μL of human TruStain FcX™ (Biolegend, cat # 422301) Fc Blocking reagent was added and incubated for 10 min at 4 °C. The antibody pool was prepared using 1 µg of single-cell TotalSeq™—A025x anti-human Hashtag antibody (Biolegend). To maximize performance, the antibody pool was centrifuged at 14,000× *g* at 2–8 °C for 10 min before adding to the single-cell suspension and incubating for 30 min at 4 °C. Cells were washed threefold with 1 mL cell staining buffer, spun for 5 min at 350× *g* at 4 °C and resuspended in 500 μL of PBS in 1.5 mL tubes. Specimen samples were pooled from all patients and stored on ice. Before sorting, 1 μg/mL of propidium iodide was added and incubated at room temperature for 5 min. Cells were passed through a 70 μm cell strainer or nylon meshes onto a new tube and loaded into the cell sorter. Cell sorting was performed with a four-laser (405 nm, 488 nm, 561 nm, 640 nm) BD FACSAria III high-speed cell sorter with a 70 µm, 85 µm, and 100 µm nozzle. Living cells were collected in a new 1.5 mL tube containing 1 mL of PBS, pelleted by centrifugation and resuspended in fresh PBS.

### 4.4. Library Preparation

Library preparation of DNA samples, in order to be compatible with the Illumina sequencer (10x Genomics Inc., San Diego, CA, USA), was performed with the Illumina 10x Genomics sequencing workflow in order to generate droplet-based scRNA-seq data. Samples were prepared for primary PDL, cultured PDL cells in serum-containing medium (PDL_M1), as well as cultured PDL cells in serum-free medium (PDL_M2). Library preparation was done according to the manufacturer’s protocol (10x Genomics, Chromium Single Cell 3′ Reagent Kits v3 and CITE-seq). First, the viability of sorted cells and the degree of separation were assessed using the Countess II system (Thermo). Subsequently, 2500 cells were used to prepare the libraries. According to the CITE-seq protocol, a specific HTO primer had been added to the cDNA amplification step. In the subsequent cDNA purification, the supernatant was used for HTO library preparation, while the bead-bound DNA was used for cDNA library preparation. Sequencing was performed on the NovaSeq 6000 system using an SP flow cell. According to 10x Genomics guidelines, read lengths were 28/8/0/91.

### 4.5. Sequencing and Demultiplexing

All libraries were pooled and sequenced on a NovaSeq sequencing system with an SP flow cell, adhering to the following run parameters: 28 cycles for read 1, 91 cycles for read 2, and 8 cycles for index 1. The instrument’s standard sequencing workflow mainly consists of template generation, imaging, and base calling with RTA. The binary sequencing data were then demultiplexed and converted to fastq format using bcl2fastq v2.20.0.422 (https://support.illumina.com/sequencing/sequencing_software/bcl2fastq-conversion-software.html; access date: 4 December 2021)) without allowing for any index-based mismatch (--barcode-mismatches 0). This precaution was taken to consider the small edit distance between two of the sample barcodes used in library preparation. The sequencing resulted in average sample sizes of 72 M, 157.5 M, and 82 M sequencing reads for PDL, PDL_M1, and PDL_M2, respectively, and 56.5 M, 88.5 M, and 102.5 M reads for the corresponding HTO libraries. This reads distribution was predetermined based on a calculated pooling ratio, derived from a preliminary shallow sequencing experiment and subsequent saturation analysis to reach the sample-specific number of reads required for optimal saturation at the gene expression and HTO levels for each sample.

### 4.6. Cellranger Primary Analysis

The CellRanger analysis pipeline (https://www.10xgenomics.com/support/software/cell-ranger/downloads/previous-versions) (v3.1.0-access date: 23 April 2023) from 10x was used to perform the standard scRNA-seq basic analysis. The pipeline workflow includes the following steps: (1) alignment to the human genome reference sequence GRCh38 (preprocessed release 95—downloaded from the 10x Genomics website), (2) cell inference, (3) saturation analysis, and (4) various QC metrics. Starting from 1250, 2500, and 2500 initially set cells for PDL, PDL_M1, and PDL_M2, respectively, cell recovery rates of 21% (262 cells), 16.2% (404 cells), and 10.7% (267 cells) were achieved. In addition, average sequencing depths of 276K, 390K, and 300K reads per cell were received, respectively, which is significantly higher than the recommended average depth. Finally, for the HTO libraries, 5K, 63K, and 59K usable HTO reads per cell were produced (10x recommendation is 5K sequenced reads per cell). All samples reached a saturation level of approximately 98% in terms of sequencing depth.

### 4.7. Downstream Analysis with Seurat

#### 4.7.1. Data Integration and Cleaning

The Cellranger software (version 4.6) was used to create single-cell gene expression matrices for downstream analysis, applying its standard filtering process to distinguish between GEMs containing cells and those either empty or containing ambient RNA. The Seurat package (v3.9.9.9008) was then used to further process the filtered gene expression matrices (UMI counts per gene per cell) and de-multiplex the donor samples based on the hash information (HTO). For this purpose, an algorithm applying Seurat’s HTODemux function was used which sets the quantile parameter to 99.9% and otherwise uses standard parameters [35]. Samples were integrated using Seurat’s standard integration workflow and analyzed independently.

#### 4.7.2. Quality Checks

Analysis of the scRNA-seq data started with an initial quality control assessing standard quality parameters to evaluate cell status in individual samples. To enhance data quality, noisy cells, defined by mitochondrial or ribosomal loads comprising more than 25% or 50% of the detected transcriptome, respectively, were excluded. Cells containing fewer than 150 genes were also sorted out. Metrics comprising mitochondrial load in each cell (mt genes) as marker for cell degradation and cell death, ribosomal load (ribo genes) as indicator for cell cycle, the number of genes detected per cell (n feature), and number of transcripts (n count) were determined in order to eliminate low-quality cells falsifying analyses. Doublets were filtered out, identified by an excess number of detected genes and transcripts within a single barcode. Absence of cells indicating false positives, dying cells, or debris were removed. The cut-off threshold was set at 20, removing all cells exceeding this threshold from further analysis. This further selection retaining only high-quality cells, took place after integration, as the poor quality cells were also used during integration to give the algorithm additional complexity. Cells with missing hash identity were retained for downstream analyses to enhance clustering performance and subsequent steps. Subsequently, HTO (hashtagging) demultiplexing was performed, with each sample barcoded and linked to HTO signals. The strongest signal was used as the donor identity using the HTODemux method with a quantile of 99.9%. Cell identities were categorized into singlets, cells with negative signals, and doublets. Singlets, representing single cells with statistically significant unique identity from one donor, were further processed for analysis. Negatives referred to singlets with either no or non-significant hashtagging, while doublets contain multiple cells. Finally, all identified noisy and biased data, including doublets, cells with fewer than 150 genes, cells with more than 25% mitochondrial genes, and cells with 50% ribosomal genes, were removed.

#### 4.7.3. Dimensionality Reduction and Clustering

Dimensionality reduction via standard PCA analysis was initially performed, with the first 20 dimensions selected for the PDL batch after consulting the elbow plot (principal components against standard deviation). Subsequently, UMAP dimensionality reduction was performed based on its implementation in the wot package using the RunUMAP function with the mentioned dimensions and otherwise the default parameters [36]. The subsequent clustering of the cells was carried out using an SNN (Shared Nearest Neighbor) clustering algorithm based on modularity optimization via the FindClusters function. This process entailed calculating the k-nearest neighbors of the cell, constructing the SNN graph and then optimizing a modularity function to determine the clusters based on a given resolution. A clustering resolution of 0.25 was chosen for integrated PDL samples to avoid overclustering.

#### 4.7.4. Differential Expression Analyses

Differential expression analyses between clusters were performed with FindAllMarkers, which implements the nonparametric Wilcoxon rank sum test. To identify conserved markers between donors, the FindConvervedMarkers function was used to calculate the differential markers for each specific cluster and each donor present in the cluster compared to all other clusters and then combine the markers of all donors that have a combined significant meta-*p*-value. The standard minimum method from the R package metap was used to combine the *p*-values. For each cluster, only donors contributing more than two cells in the cluster were considered to obtain reliable conclusions. To identify genes with high specificity, the discrimination analysis method implemented with GeneSorteR was applied [37].

### 4.8. Pseudotemporal Trajectory Inference

The SCORPIUS package was used for pseudotemporal trajectory inference of single-cell data to determine the order of cells along developmental trajectories [38]. To construct trajectories, a dimensionality reduction was performed with the reduce_dimensionality method of the package, using the Spearman correlation for the distance parameter and selecting three dimensions. Then, the infere_trajectory method was run with the default parameters. The draw_trajectory_plot method was applied to plot the trajectory.

## 5. Conclusions

In this study, we have employed cutting-edge scRNA-seq technology to gain deeper insight into the genetic profile of the PDL. Our exploration has uncovered the intricate cellular populations within the PDL in their highly organized complexity and individuality, unveiling distinct genetic signatures and cell clusters. Notably, we have deciphered both inherent subpopulations and those influenced by contextual aspects, shedding light on the dynamic nature of the PDL.

Furthermore, our findings underscore the profound impact of in vitro transitions and serum-containing cell culture on the genetic features of PDL cells. This revelation not only deepens our comprehension of the cellular architecture of the PDL but also underscores the importance of environmental factors in shaping its characteristics.

By elucidating these phenomena, our study lays a solid foundation for future investigations aimed at unraveling the underlying determinants of these observations. These insights promise to advance our understanding of the PDL’s fundamental dynamics and pave the way for further research in this field.

## Data Availability

The raw data supporting the conclusions of this article will be made available by the authors on request.
